# Kaplan and Sadock's Synopsis of Psychiatry: Behavioral Sciences/Clinical Psychiatry, 10^th^ edition

**Published:** 2009

**Authors:** Sujit Sarkhel

**Affiliations:** Room No. 14, Men's Hostel No. 1, Central Institute of Psychiatry, Ranchi - 834 006, India. E-mail: suj_doc@yahoo.com

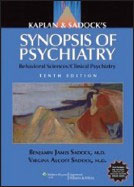



The current edition of “Synopsis of Psychiatry” is the third in this series (the eighth edition being the first) to adorn my bookshelf, and like the previous ones, does adequate justice to the expectations of the mental health fraternity. The purpose of this series is clearly spelt out: To provide a condensed and a more “manageable” version of the much more voluminous “Comprehensive Textbook of Psychiatry” by the same editors. This purpose has been undoubtedly accomplished. The width of coverage has not been compromised for the sake of brevity.

The opening chapter deals effectively with the most important aspect of psychiatry: The patient-doctor relationship. Subsequent sections deal with basic neurosciences and psychosocial sciences. The newly added chapter on neurogenetics is lucidly written and useful.

The chapters on various psychiatric disorders follow the pattern of previous editions: Evolution of the concept, epidemiology, etiology, diagnostic and clinical features, differential diagnosis and management. Well-chosen case vignettes add to the understanding of various disorders. The ICD-10 diagnostic criteria (which are used more commonly in our country) deserved a more prominent place (vis a vis the DSM-IV-TR criteria) rather than a passing mention at the tail-end of each chapter.

Throughout the text, Tables, Figures and photos have been freely used to enhance understanding as well as interest of the readers. However, some of the color plates are out of place. For example, the plate depicting brain regions involved in mood disorders is placed in the chapter on schizophrenia.

A new chapter on “Psychiatric emergencies in children” has been added in the section of psychiatric emergencies. Well in keeping with the recent advances, the chapters on genetic counseling and dialectical behavior therapy have been welcome additions to the psychotherapy section. Interpersonal therapy, which formed a part of the chapter on brief psychotherapy in the previous edition, has been dealt with separately, laying special emphasis on delivering the therapy in a group format.

The section on psychopharmacology incorporates all new molecules that have been approved for treatment of various psychiatric conditions-newer anticonvulsants, phosphodiasterase-5 inhibitors, memantine and so on. A chapter on “other methods of brain stimulation” has been added. In this chapter, only the section on deep brain stimulation is new whereas several other therapeutic modalities of historical interest like insulin coma therapy have been deleted. Arrangement of drugs based on their mechanisms of action rather than their indications is one of the best features of this book, because, with the advent of latest research, newer indications of older molecules keep emerging.

One of the major additions in the child psychiatry section is the chapter on “childhood anxiety disorders” and impact of terrorism on children. Both are lucid, well-written and up to date. Another important addition in the chapter of end of life care includes a section on physician-assisted suicide. However, this section does not provide much additional information in comparison with the previous edition.

In the chapters dealing with major psychiatric disorders, treatment algorithms (as per available guidelines) could have been included. In this era of evidence-based medicine, a chapter on evidence-based psychiatry is conspicuous by its absence. In an effort to keep up with massive advances in the field of biological psychiatry without increasing the volume of the text, several areas related to conceptual evolution of psychiatric disorders (especially, psychosocial perspectives) have taken a back seat. This is unfortunate, not only because it narrows the perspective of the psychiatry residents but also may pose difficulty in exams as questions from these areas are frequently asked.

Overall, this book is worth the money and a must in the bookshelves of all mental health trainees and professionals. Undergraduate medical students may, however, find the book a little difficult to comprehend.

